# Adsorption Features of Tetrahalomethanes (CX_4_; X = F, Cl, and Br) on *β*_12_ Borophene and Pristine Graphene Nanosheets: A Comparative DFT Study

**DOI:** 10.3390/molecules28145476

**Published:** 2023-07-18

**Authors:** Mahmoud A. A. Ibrahim, Amna H. M. Mahmoud, Nayra A. M. Moussa, Gamal A. H. Mekhemer, Shaban R. M. Sayed, Muhammad Naeem Ahmed, Mohamed K. Abd El-Rahman, Eslam Dabbish, Tamer Shoeib

**Affiliations:** 1 Computational Chemistry Laboratory, Chemistry Department, Faculty of Science, Minia University, Minia 61519, Egypt; a.mahmoud@compchem.net (A.H.M.M.); n.moussa@compchem.net (N.A.M.M.); gmekhemer@mu.edu.eg (G.A.H.M.); 2 School of Health Sciences, University of KwaZulu-Natal, Westville Campus, Durban 4000, South Africa; 3 Department of Botany and Microbiology, College of Science, King Saud University, P.O. Box 2455, Riyadh 11451, Saudi Arabia; shmohamed@ksu.edu.sa; 4 Department of Chemistry, The University of Azad Jammu and Kashmir, Muzaffarabad 13100, Pakistan; drnaeem@ajku.edu.pk; 5 Department of Chemistry and Chemical Biology, Harvard University, 12 Oxford Street, Cambridge, MA 02138, USA; kabdelazim@gmwgroup.harvard.edu; 6 Department of Chemistry, The American University in Cairo, New Cairo 11835, Egypt; emoustafa@aucegypt.edu

**Keywords:** tetrahalomethanes, graphene nanosheet, borophene nanosheet, DFT

## Abstract

The potentiality of the *β*_12_ borophene (*β*_12_) and pristine graphene (GN) nanosheets to adsorb tetrahalomethanes (CX_4_; X = F, Cl, and Br) were investigated using density functional theory (DFT) methods. To provide a thorough understanding of the adsorption process, tetrel (XC-X_3_∙∙∙*β*_12_/GN)- and halogen (X_3_C-X∙∙∙*β*_12_/GN)-oriented configurations were characterized at various adsorption sites. According to the energetic manifestations, the adsorption process of the CX_4_∙∙∙*β*_12_/GN complexes within the tetrel-oriented configuration led to more desirable negative adsorption energy (*E*_ads_) values than that within the halogen-oriented analogs. Numerically, *E*_ads_ values of the CBr_4_∙∙∙Br1@*β*_12_ and T@GN complexes within tetrel-/halogen-oriented configurations were −12.33/−8.91 and −10.03/−6.00 kcal/mol, respectively. Frontier molecular orbital (FMO) results exhibited changes in the *E*_HOMO_, *E*_LUMO_, and *E*_gap_ values of the pure *β*_12_ and GN nanosheets following the adsorption of CX_4_ molecules. Bader charge transfer findings outlined the electron-donating property for the CX_4_ molecules after adsorbing on the *β*_12_ and GN nanosheets within the two modeled configurations, except the adsorbed CBr_4_ molecule on the GN sheet within the tetrel-oriented configuration. Following the adsorption process, new bands and peaks were observed in the band structure and density of state (DOS) plots, respectively, with a larger number in the case of the tetrel-oriented configuration than in the halogen-oriented one. According to the solvent effect affirmations, adsorption energies of the CX_4_∙∙∙*β*_12_/GN complexes increased in the presence of a water medium. The results of this study will serve as a focal point for experimentalists to better comprehend the adsorption behavior of *β*_12_ and GN nanosheets toward small toxic molecules.

## 1. Introduction

Two-dimensional (2D) nanomaterials have recently been of universal interest owing to their outstanding chemical and physical properties [1,2,3,4]. As a premier developed 2D material, pristine graphene (GN) was regarded as the most intriguing star in the realm of materials science [5,6,7,8,9,10,11]. GN-based materials were denoted with unique features, including a high specific surface area [12], quantum Hall effect [13], high thermal conductivity [14], and ambipolar electric field effect [15]. Such properties shed light on their vast-ranging applications, like energy storage [16,17], drug delivery [18,19,20,21], spintronics [22], and catalysis [23]. Because of their low electronic noise, GN-based materials were also announced as an appealing candidate for adsorbing chemical systems [6,24].

Following the astonishing discovery of GN sheets, considerable research has been directed to develop various 2D materials, including antimonene [25], hexagonal boron nitride (h-BN) [26], bismuthine [27], silicene [28,29], and borophene [30,31]. In the parallel area, borophene, a 2D boron sheet, was announced with extraordinary properties, like electron mobility, anisotropic properties, superconductivity, and its phonon-mediated form [32,33,34]. Borophene was successfully fabricated on a single surface of Ag(111) under ultrahigh-vacuum conditions [30,31]. Different borophene phases were observed at various deposition temperatures using a high-resolution scanning tunneling microscope (STM), such as the striped, *β*_12_, and χ_3_ phases [31]. The puckered shape and metallic characteristics of the striped phase led to its utility in various potential applications for metal ion storage and electric conduction [35]. Compared with the striped phase, the preferable stability of the *β*_12_ and the χ_3_ phases, with planar shapes having hexagonal and triangular vacancies, was demonstrated [36,37]. Because of its structure with a hexagonal vacancy, borophene was utilized in adsorbing gas molecules [10,38,39,40].

An upsurge in interest has recently been oriented toward investigating the utility of borophene and GN in the detection of gas molecules, like NO, CO, NO_2_, CO_2_, CS_2_, and NH_3_ molecules [40,41,42,43]. Halomethanes are known for being toxic molecules [44,45,46]; however, scant attention has been directed toward exploring novel nanomaterials for adsorbing them. Using density functional theory (DFT), the adsorption of tetrahalomethanes CX_4_ (X = F, Cl, and Br) was studied on carbon nanotubes [47] and GN nanosheets [48]. Nevertheless, no comparative study provided a full insight into the adsorption process of the tetrahalomethanes via all their possible oriented configurations on the surface of the borophene and GN nanosheets.

**Figure 1:**
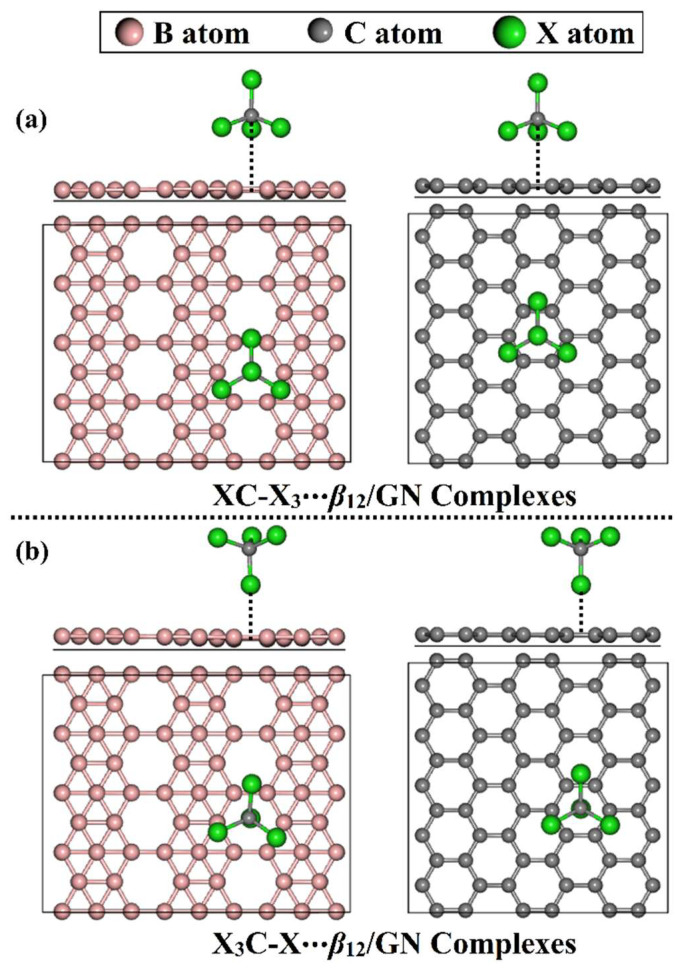
Side and top representations of the CX_4_∙∙∙*β*_12_/GN complexes (where X = F, Cl, and Br) within (**a**) tetrel (XC-X_3_)- and (**b**) halogen (X_3_C-X)-oriented configurations.

Herein, the adsorption features of tetrahalomethanes (CX_4_, where X = F, Cl, and Br) on the *β*_12_ borophene (*β*_12_) nanosheet were unveiled and compared with those with the utilization of the GN nanosheet as the starting 2D nanomaterial. The CX_4_∙∙∙*β*_12_/GN complexes were selectively studied within tetrel (XC-X_3_)- and halogen (X_3_C-X)-oriented configurations (Figure 1) using various density functional theory (DFT) method. Initially, geometry relaxation of the potential binding modes of the two suggested configurations and their corresponding adsorption energies were first carried out. Additionally, to assess the change in the electronic characteristics of the studied 2D nanosheets following the adsorption of the CX_4_ molecules, Bader charge, electronic band structure, and density of state (DOS) calculations were conducted. Further, the solvent effect on the adsorption energy of the studied complexes was evaluated. The obtained results would be an informative base for the utilization of the *β*_12_ and GN in adsorbing small molecules, such as tetrahalomethanes.

## 2. Results and Discussion

### 2.1. Geometric Structures

**Figure 2:**
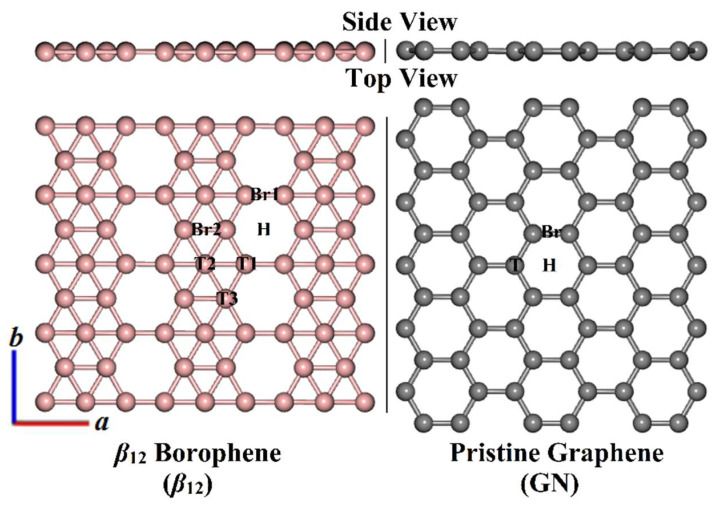
Side and top perspectives of the relaxed structures of 3 × 4 × 1 *β*_12_ borophene (*β*_12_) and 6 × 5 × 1 pristine graphene (GN) with the studied adsorption sites. The boron and carbon atoms are represented by pink and gray colors, respectively. Top, hollow, and bridge adsorption sites are referred to as T, H, and Br, respectively.

*β*_12_ and GN structures were modeled and relaxed before the adsorption process of the tetrahalomethanes. The optimized *β*_12_ and GN structures are presented in Figure 2. The obtained equilibrium lattice constants for the primitive cells of the *β*_12_ nanosheet were *a* = 5.06 Å and *b* = 2.93 Å. For the GN nanosheet, the equilibrium lattice constants were *a* = *b* = 2.47 Å. The current findings are consistent with earlier research [30,49,50]. On the *β*_12_ optimized structure, six adsorption sites were detected, comprising three top (T1, T2, and T3), two bridge (Br1 and Br2), and one hollow (H) sites (Figure 2). Looking at the GN surface, three adsorption sites, namely the top (T), bridge (Br), and hollow (H) sites, were noticed (Figure 2).

### 2.2. Adsorption Energy Calculations

**Figure 3:**
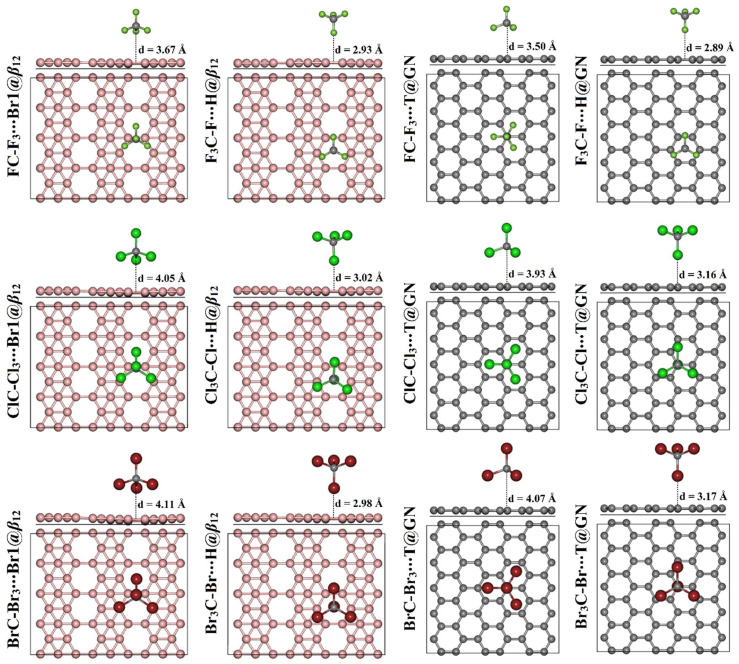
Side and top perspectives of the relaxed CX_4_∙∙∙*β*_12_/GN complexes (where X = F, Cl, and Br) at the most preferable adsorption sites within tetrel (XC-X_3_)- and halogen (X_3_C-X)-oriented configurations. Equilibrium distances (d) are in Å. The boron, carbon, fluorine, chlorine, and bromine atoms are defined by pink, gray, pale green, green, and red colors, respectively.

The adsorption of tetrahalomethanes CX_4_ (where X = F, Cl, and Br) on the surfaces of *β*_12_ and GN was investigated at different adsorption sites within the tetrel (XC-X_3_)- and halogen (X_3_C-X)-oriented configurations. The adsorption energies and the corresponding equilibrium distances of all relaxed CX_4_∙∙∙*β*_12_/GN complexes were calculated and are summarized in Table 1. Figure S1 illustrates all relaxed complexes. The relaxed CX_4_∙∙∙*β*_12_/GN complexes at the most energetically preferable adsorption sites are displayed in Figure 3.

For the adsorption process of the CX_4_ on the *β*_12_ nanosheet within the tetrel-oriented configuration, the BrC-Br_3_∙∙∙*β*_12_ complexes had the most significant *E*_ads_ values, followed by the ClC-Cl_3_∙∙∙*β*_12_, then the FC-F_3_∙∙∙*β*_12_ complexes (Table 1). Numerically, the *E*_ads_ of the BrC-Br_3_∙∙∙, ClC-Cl_3_∙∙∙, and FC-F_3_∙∙∙Br1@*β*_12_ complexes were −12.33, −7.74, and −4.46 kcal/mol, respectively. These findings were in accord with a prior study, indicating that the adsorption energies increased with the increasing atomic size of the halogen atom (decreasing the electronegativity of the halogen atom) [51]. It is worth noting that the most preferred complex was the BrC-Br_3_∙∙∙Br1@*β*_12_ complex, with an *E*_ads_ value of −12.33 kcal/mol and an equilibrium distance of 4.11 Å. In line with the tetrel-oriented configuration, energetic manifestations of the halogen-oriented complexes (i.e., X_3_C-X∙∙∙*β*_12_) showed the existence of a direct correlation between the adsorption energy and the atomic size of the halogen atom. Apparently, the H@*β*_12_ site was the most appropriate adsorption site for adsorbing the X_3_C-X molecules in the halogen-oriented configuration. Moreover, the Br_3_C-Br∙∙∙H@*β*_12_ complex had the most prominent *E*_ads_ with a value of −9.00 kcal/mol at an equilibrium distance of 2.98 Å. The efficiency of the *β*_12_ nanosheet to adsorb the CX_4_ molecules was more significant in the tetrel-oriented configuration than in the halogen-oriented one (Table 1). For instance, the *E*_ads_ values of the adsorption of the CBr_4_ at the Br1@*β*_12_ site were −12.33 and −8.91 kcal/mol in the tetrel- and halogen-oriented configurations, respectively.

For the adsorption of CX_4_ on the GN nanosheet, all complexes showed negative *E*_ads_ values, confirming the occurrence of the adsorption process. Similar to the CX_4_∙∙∙*β*_12_ complexes, the preferentiality of the adsorption process of the CX_4_ molecules on the GN nanosheet increased upon the following order X = F < Cl < Br. Obviously, the T@GN site had the highest tendency for adsorbing the studied tetrahalomethanes on the GN sheet, exhibiting significant *E*_ads_ values. Numerically, the ClC-Cl_3_∙∙∙ and Cl_3_C-Cl∙∙∙T@GN complexes had *E*_ads_ values of −7.32 and −4.22 kcal/mol, respectively (Table 1). 

For all CX_4_∙∙∙*β*_12_/GN complexes, the obtained *E*_ads_ values ranged from −2.46 to −12.33 kcal/mol, demonstrating the occurrence of physisorption processes. The latter observation was in line with the literature, which reported the adsorption energy of CH_4_∙∙∙, CF_4_∙∙∙, and CCl_4_∙∙∙GN complexes with values of −1.61, −3.46, and −8.99 kcal/mol, respectively [52]. While the adsorption of the CH_4_ molecule on the borophene nanosheet exhibited a small *E*_ads_ value of −2.54 kcal/mol and was accordingly documented as a physisorption process [53]. For a given type of halogen, the *β*_12_ nanosheet showed more affinity to adsorb the CX_4_ molecules than the GN nanosheet, which can be attributed to the lower electronegativity of boron relative to carbon and, hence, a lower electronegativity difference compared with that of the halogen.

Besides, the adsorption of the tetrahalomethanes became more favorable by decreasing the electronegativity of the halogens in the following order CF_4_∙∙∙ > CCl_4_∙∙∙ > CBr_4_∙∙∙*β*_12_/GN, which was accompanied by a lower electronegativity difference in the case of boron compared with carbon atoms. The favorability of the adsorption process within the tetrel-oriented configuration might be attributed to the contribution of the three halogen atoms of XC-X_3_ molecules to the overall interaction. 

### 2.3. Frontier Molecular Orbital (FMO) Calculations

In order to comprehend the effect of the adsorption process on the electronic characteristics of the examined systems, the energies of the highest occupied molecular orbitals (*E*_HOMO_), the lowest unoccupied molecular orbitals (*E*_LUMO_), and the energy gap (*E*_gap_) values were assessed. Table 2 shows data of the *E*_HOMO_, *E*_LUMO_, and *E*_gap_ values of the investigated systems before and following the adsorption process. 

According to the data in Table 2, notable differences in the *E*_HOMO_, *E*_LUMO_, and *E*_gap_ values were observed for the studied systems before and following the adsorption process. For instance, in the tetrel-oriented configuration, the *E*_HOMO_ value of the BrC-Br_3_∙∙∙Br1@*β*_12_ complex was −2.544 eV, whereas the pure *β*_12_ nanosheet had an *E*_HOMO_ value of −2.875 eV (Table 2). Moreover, the *E*_gap_ values of all CX_4_ molecules, *β*_12_, and GN nanosheets were altered, confirming the occurrence of adsorption processes. For example, the pure *β*_12_ nanosheet had an *E*_gap_ value of −0.626 eV that was changed to −0.602 eV after the adsorption process within the BrC-Br_3_∙∙∙Br1@*β*_12_ complex (Table 2). 

### 2.4. Charge Transfer Calculations

The Bader charge method is a reliable appliance for determining the charge transfer over the adsorption process [54,55]. The transferred charge between the CX_4_ molecules and the 2D nanosheets within all the studied complexes was evaluated in terms of the charge transfer difference (*Q*_t_) values (Table 1). The *Q*_t_ values with negative signs remarked that the charge was shifted from the CX_4_ molecules towards the *β*_12_ and GN nanosheets, and vice versa was true for the positive *Q*_t_ values. 

Table 1 shows *Q*_t_ values with a negative sign for the CX_4_∙∙∙*β*_12_ complexes, demonstrating the ability of the inspected tetrahalomethanes to donate electrons to the *β*_12_ nanosheets within the tetrel- and halogen-oriented configurations. Notably, the *Q*_t_ values of the CX_4_∙∙∙*β*_12_ complexes within the halogen-oriented configuration generally decreased as the adsorption energies decreased (i.e., in the order CBr_4_∙∙∙ > CC1_4_∙∙∙ > CF_4_∙∙∙*β*_12_). For instance, the *Q*_t_ values of the Br_3_C-Br∙∙∙, Cl_3_C-Cl∙∙∙, and F_3_C-F∙∙∙T1@*β*_12_ complexes were −0.0654, −0.0410, and −0.0164 *e*, respectively. The reversed trend was noticed for the complexes within the tetrel-oriented configuration, outlining the noticeable contributions of the three coplanar halogen atoms to the adsorption process. For example, the *Q*_t_ values for the CF_4_∙∙∙, CCl_4_∙∙∙, and CBr_4_∙∙∙Br1@*β*_12_ complexes within the tetrel-oriented configuration were −0.0313, −0.0283, and −0.0263 *e*, respectively.

The *Q*_t_ values of the CX_4_∙∙∙GN complexes within the tetrel- and halogen-oriented configurations showed similar trends to the CX_4_∙∙∙*β*_12_ complexes, except for the CBr_4_∙∙∙GN complexes within the former configuration that had positive *Q*_t_ values. For the latter complexes, the electron-accepting property increased in the following order, H@GN < Br@GN < T@GN adsorption sites, and was confirmed with positive *Q*_t_ values of 0.0070, 0.0023, and 0.0036 *e*, respectively. 

**Figure 4:**
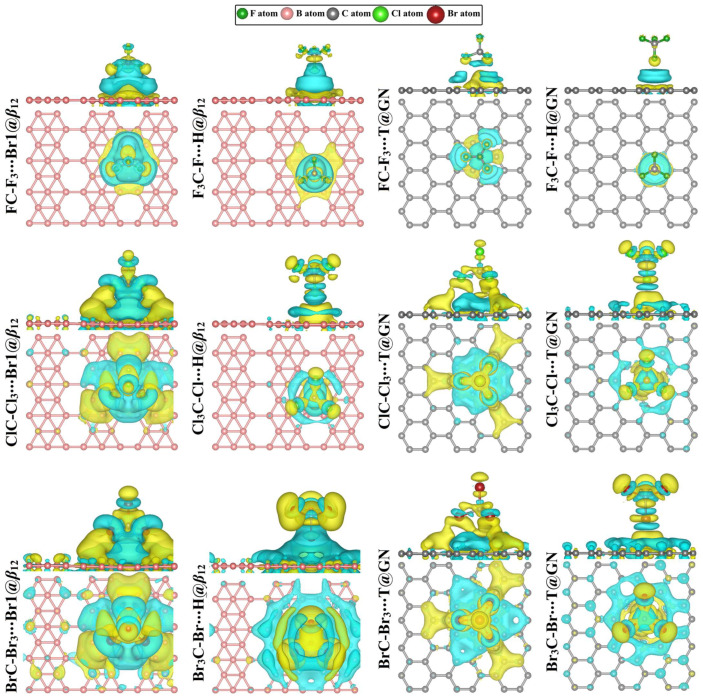
Charge density difference (∆*ρ*) maps of the relaxed CX_4_∙∙∙*β*_12_/GN complexes (where X = F, Cl, and Br) at the most preferable adsorption sites within tetrel (XC-X_3_)- and halogen (X_3_C-X)-oriented configurations. Regions with cyan and yellow colors refer to depletion (negative) and accumulation (positive) charges, respectively. The isosurface values for the CX_4_∙∙∙*β*_12_ and ∙∙∙GN were set to be 3.08 × 10^−5^ and 5.0 × 10^−5^ *e*/Å^3^, respectively.

Charge density difference (∆*ρ*) maps were generated in order to investigate the distribution of the charge within the relaxed CX_4_∙∙∙*β*_12_/GN complexes at the most preferable adsorption sites, and the maps are provided in Figure 4. As demonstrated in Figure 4, the electron depletion and accumulation regions (i.e., cyan- and yellow-colored regions, respectively) revealed the distribution of the charge between the tetrahalomethanes and the investigated 2D nanosheets. Apparently, the most remarkable electron-accumulated region was observed for the CBr_4_∙∙∙*β*_12_/GN complexes, demonstrating the further ability of CBr_4_ molecules to be adsorbed on the studied 2D nanosheets among tetrahalomethane analogs (Figure 4). 

Overall, the negative *Q*_t_ values confirmed that all the CX_4_ molecules had an electron-donating character except for the CBr_4_∙∙∙GN complexes within the tetrel-oriented configuration. According to the *Q*_t_ values, the amount of charge transferred from the tetrahalomethanes to the *β*_12_ nanosheet was more significant than that of the GN nanosheet, which was in line with the adsorption energy values. Based on ∆*ρ* maps, it was observed that the amount of the distribution charge area (colored area) increased as the electronegativity of the halogen atom decreased. For instance, the size of the distribution charge area increased as the atomic size of the halogen atom increased in the order FC-F_3_∙∙∙ < ClC-Cl_3_∙∙∙ < BrC-Br_3_∙∙∙Br1@*β*_12_ complexes. 

Given that the *Q*_t_ values of the CX_4_∙∙∙*β*_12_/GN complexes within the halogen-oriented configuration generally decreased as the adsorption energies decreased, while the reversed trend was noticed for the complexes within the tetrel-oriented configuration. This implies that the halogen orientation relies on a more localized charge transfer and electronegativity difference during the binding mechanism, while the tetrel orientation is accompanied by a more distributed charge transfer binding that is more significant with large-sized bromine atoms.

### 2.5. Band Structure Calculations

To ascertain the impact of the adsorption of the CX_4_ molecules on the electronic properties of the *β*_12_ and GN nanosheets, band structure analysis was carried out for the pure and combined 2D nanosheets. Using PBE functional along the high-symmetry paths of the Brillouin zone, band structures were extracted. The Γ-Y-S-X-Γ path was chosen for the *β*_12_ nanosheet, and the Y-S-X-Γ-Y path was selected for the GN nanosheet. The band structures of the pure 2D nanosheets are demonstrated in Figure S2. 

Looking at Figure S2, a metallic character of the pure *β*_12_ surface was noted by several bands, which crossed the Fermi level along the high-symmetry path. For the pure GN surface, the existence of the Dirac point at the Fermi level announced its semiconducting property.

**Figure 5:**
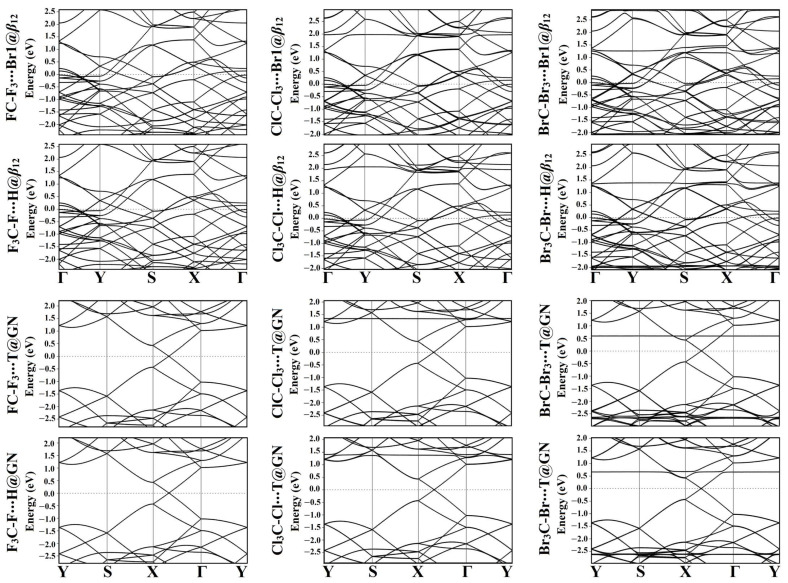
Band structure plots for the relaxed CX_4_∙∙∙*β*_12_/GN complexes (where X = F, Cl, and Br) at the most preferable adsorption sites within tetrel (XC-X_3_)- and halogen (X_3_C-X)-oriented configurations. The Fermi energy was positioned at zero energy.

Band structures of the relaxed CX_4_∙∙∙*β*_12_/GN complexes at the most preferable adsorption sites are plotted in Figure 5. After the adsorption process, slight differences were noticed in the electronic band structures of the pure nanosheets, outlining the physisorption process of tetrahalomethanes on the pure nanosheets (Figure 5).

For the adsorption of CF_4_ molecules, insignificant changes were denoted in the electronic band structures of the *β*_12_ nanosheet. Upon adsorbing CCl_4_ and CBr_4_ molecules, further new bands appeared in the band structures of the combined nanosheets compared with the pure analogs. Such new bands remarked the adsorption of the CCl_4_ and CBr_4_ molecules on the *β*_12_ nanosheet. Illustratively, the CBr_4_∙∙∙*β*_12_ complexes displayed a new conduction band at 1.35 eV and new valence bands at −0.60 and −2.00 eV. It was also observed that the bands shifted towards the Fermi level in the case of the complexes within the tetrel-oriented configuration more than the halogen-oriented analog. For instance, the adsorption of CCl_4_ at the Br1@*β*_12_ and H@*β*_12_ within the tetrel- and halogen-oriented configurations led to the appearance of a conduction band at around 2.70 and 2.15 eV, respectively. This observation demonstrated the higher favorability of the adsorption process within the former configuration than the latter one.

Similar to the *β*_12_ nanosheet, the CF_4_ molecules had a neglected effect on the band structure of the pure GN surface (Figure 5). Besides, the band structures of the CCl_4_∙∙∙ and CBr_4_∙∙∙GN complexes showed many new valence and conduction bands, confirming the higher potentiality of the GN nanosheet to adsorb these molecules compared with CF_4_ molecules. For instance, in the CBr_4_∙∙∙GN complexes, a new conduction band appeared at 0.60 and 0.67 eV, respectively, while in the valence region, many valence bands appeared at −2.40 eV and then ranged from −2.62 to −2.65 eV (Figure 5). It can be seen that the valence and conduction bands in the CX_4_∙∙∙GN complexes shifted towards the Fermi level as the atomic size of the halogen atom increased, demonstrating a favorable adsorption process. For example, the valence band at around −2.55 eV in the FC-F_3_∙∙∙T@GN complex shifted to −2.60 eV in the ClC-Cl_3_∙∙∙T@GN complex, and then to −2.65 eV in the BrC-Br_3_∙∙∙T@GN complex (Figure 5).

Summing up, the band structures of the *β*_12_ nanosheet demonstrated more new bands after adsorbing the CX_4_ molecules than those of the GN nanosheet. The latter affirmation indicated the further desirability of the adsorption process on the *β*_12_ nanosheet than the GN nanosheet. The obtained findings were in line with the adsorption energy affirmations. The appearance of the new bands after the adsorption process indicated the overlap of the bands of the adsorbent and substrate, confirming the interaction between the CX_4_ molecule and the studied 2D nanosheet. Further, the number of the new bands increased as the electronegativity of the halogen atom decreased. Illustratively, the CBr_4_∙∙∙*β*_12_/GN complexes, which exhibited the highest negative adsorption energy, showed the largest number of new bands among the other complexes (Figure 5).

### 2.6. Density of State Calculations

**Figure 6:**
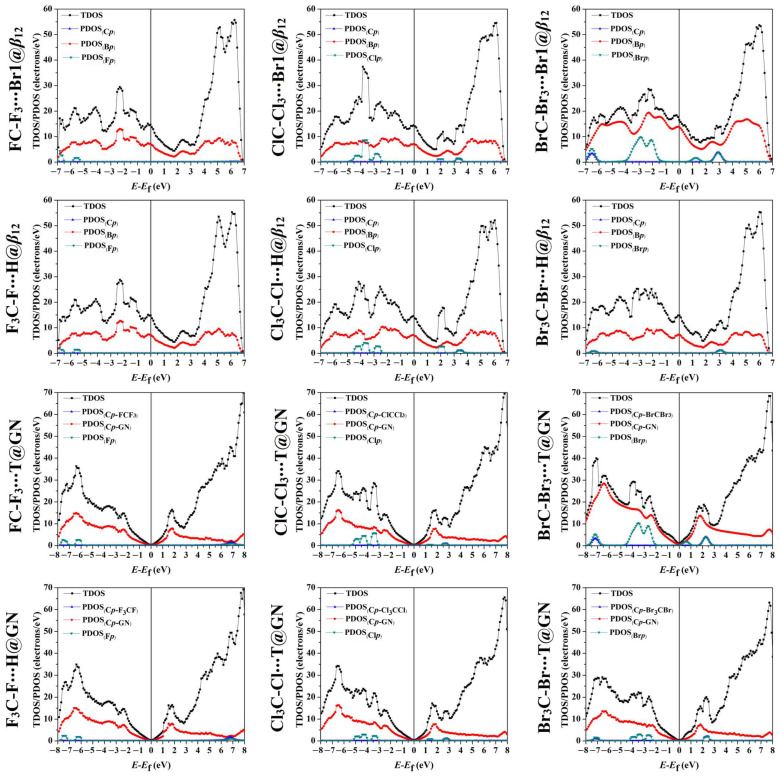
Total density of state (TDOS) plots for the relaxed CX_4_∙∙∙*β*_12_/GN complexes (where X = F, Cl, and Br) at the most preferable adsorption sites within tetrel (XC-X_3_)- and halogen (X_3_C-X)-oriented configurations. The projected density of state (PDOS) with the contribution of B*_p_*, C*_p_*_-GN_, $C_{P-{CX}_{4}}$, and $X_{P-{CX}_{4}}$. The Fermi energy was set at zero energy.

The total density of state (TDOS), together with the projected density of state (PDOS), were extracted for pure and combined 2D nanosheets to truly comprehend the impact of the adsorption process on the electronic characteristics of the 2D nanosheets (Figure S3). TDOS and PDOS plots of the most favorable complexes are shown in Figure 6. 

The PDOS plots with the contribution of the *p*-orbital of B, C, and X atoms within the studied complexes were plotted in the energy range from −7.00 to 7.00 eV for *β*_12_ and from −8.00 to 8.00 eV for GN.

As shown in Figure 6, intense and feeble peaks were observed for the contributions of the PDOS of the $X_{P-{CX}_{4}}$ and $C_{P-{CX}_{4}}$, respectively, to the TDOS of all the studied complexes. Accordingly, the halogens and carbon atoms of the CX_4_ molecules exhibited major and minor roles within the adsorption process on the 2D nanosheets, respectively. 

For example, the contribution of Cl*_p_* to the CCl_4_∙∙∙*β*_12_ and ∙∙∙GN complexes within the tetrel-oriented configuration were found in the valence region ranging from −2.50 to −4.70 eV and −3.00 to −5.10 eV, respectively. At the same time, the contribution of Cl*_p_* also appeared in the conduction regions from 1.70 to 2.30 eV and; 3.00 to 3.60 eV for the adsorption over the *β*_12_ nanosheet and between 2.50 and 3.00 eV for the GN analog. Within the halogen-oriented configuration, the Cl*_p_* peaks of the adsorbed CCl_4_ molecule on the *β*_12_ and GN nanosheets were noticed in the valence region between −2.40 and −4.50 eV and −3.00 and −5.00 eV, respectively. In the conduction region, the contribution of the Cl*_p_* of the adsorbed CCl_4_ molecule on the *β*_12_ and GN nanosheets were found in the energy ranges of 1.80–2.50 and 3.00–4.00 eV, and 1.10–1.60 and 2.50–3.10 eV, respectively. 

Notably, hybridizations between the *p*-orbital of the 2D nanosheets and the *p*-orbital of the CX_4_ molecules were also observed, revealing the occurrence of the adsorption process (Figure 6). For instance, an overlap between the PDOS_(B*p*)_ and the PDOS_(Cl*p*)_ appeared in the range from −3.50 to −3.90 eV in the CCl_4_∙∙∙Br1@*β*_12_ complex within the tetrel-oriented configuration, affirming the ability of the *β*_12_ nanosheet to adsorb the CCl_4_ molecule. The latter observation was consistent with the *E*_ads_ of the CCl_4_∙∙∙Br1@*β*_12_ complex with a value of −7.74 kcal/mol (Table 1). While in the CCl_4_∙∙∙H@*β*_12_ complex within the halogen-oriented configuration, a small overlap between the PDOS_(B*p*)_ of the *β*_12_ nanosheet and the PDOS_(Cl*p*)_ of the CCl_4_ molecule was noticed in the conduction region from 1.80 to 2.20 eV. This finding was in agreement with the small *E*_ads_ value of −5.58 kcal/mol.

From the DOS outlines, halogens had the dominant role in the adsorption of the CX_4_ molecules on the *β*_12_ and GN nanosheets within the modeled configurations. In line with the adsorption-energy and band structure findings, the DOS plots revealed the favorability of the *β*_12_ nanosheet over the GN analog to adsorb the tetrahalomethanes.

### 2.7. Solvent Effect Calculations

To speculate the effect of the solvent on the adsorption process within the CX_4_∙∙∙*β*_12_/GN complexes, the adsorption energy was evaluated in the presence of a water solvent. Afterwards, the solvent effect ($E_{ads}^{solventeffect}$) energy was computed for the most preferable complexes as the difference between the adsorption energies of the water solvent and vacuum (see the Computational Methodology section for details). The obtained $E_{ads}^{water}$ and $E_{ads}^{solventeffect}$ values are listed in Table 3.

According to the data presented in Table 3, the adsorption energies of the CX_4_∙∙∙*β*_12_/GN complexes in the water medium showed higher negative values compared with those in a vacuum. For instance, the $E_{ads}^{water}$ and $E_{ads}^{vacuum}$ values of the CBr_4_∙∙∙Br1@*β*_12_ complex within the tetrel-oriented configuration were −15.99 and −12.33 kcal/mol, respectively (Table 1 and Table 3, respectively). Subsequently, $E_{ads}^{\mathrm{solvent effect}}$ exhibited negative values, confirming the occurrence of the adsorption process in the water medium. As an illustration, the $E_{ads}^{\mathrm{solvent effect}}$ value of the CBr_4_∙∙∙Br1@*β*_12_ complex within the tetrel-oriented configuration was −3.66 kcal/mol. Similar to the energetic manifestation obtained in a vacuum, the more prevalent effect of the water solvent on the favorability of the adsorption process was ascribed to the complexes within the tetrel-oriented configuration compared with the halogen-oriented configuration. Numerically, as an example, the $E_{ads}^{\mathrm{solvent effect}}$ values of the CBr_4_∙∙∙Br1@*β*_12_ and ∙∙∙H@*β*_12_ complexes within the tetrel- and halogen-oriented configurations were −3.66 and −1.60 kcal/mol, respectively.

## 3. Computational Methods

The density functional theory (DFT) method was applied for all calculations [56,57] via the Quantum ESPRESSO 6.4.1 package [58,59]. Based on the Perdew–Burke–Ernzerhof (PBE) scheme, the electron exchange-correlation function was conducted utilizing the generalized gradient approximation (GGA) [60]. To represent the electron–core interaction, the ultrasoft pseudopotential (USPP) was employed [61]. The van der Waals interactions for all the executed computations were taken into account using the Grimme-D2 method [62]. The utilized energy cutoff and charge density cutoff values were 50 and 500 Ry, respectively. The total energy and the atomic force convergence criteria were 1 × 10^−5^ eV and 1 × 10^−4^ eV/Å, respectively. Based on the Monkhorst-Pack mesh, the 6 × 6 × 1 and 12 × 12 × 1 *k*-points grids were adopted for the first Brillouin zone sampling within the geometry relaxation and density of state calculations, respectively. The convergence was enhanced using the Marzari–Vanderbilt smearing method [63]. For preventing image–image interaction, a vacuum thickness of 20 Å was added along the *z*-direction of the *β*_12_ and GN nanosheets.

To model the adsorption of the tetrahalomethanes (CX_4_; X = F, Cl, and Br) on *β*_12_ and GN nanosheets, 3 × 4 × 1 and 6 × 5 × 1 supercells were constructed for *β*_12_ and GN nanosheets, respectively. Adsorption energies (*E*_ads_) of the CX_4_∙∙∙*β*_12_/GN complexes within tetrel (XC-X_3_)- and halogen (X_3_C-X)-oriented configurations were assessed as follows:

(1) $$E_{ads}=E_{{CX}_{4}\cdot \cdot \cdot 2D\;nanosheet}-({E_{{CX}_{4}}+E}_{2D\;nanosheet})$$

where $E_{{CX}_{4}\cdot \cdot \cdot 2D\;nanosheet}$, $E_{{CX}_{4}}$, and $E_{2D\;nanosheet}$ are the energies of complex, tetrahalomethane, and 2D nanosheet, respectively. Frontier molecular orbital (FMO) calculations were carried out to gain a better understanding of the adsorption process of CX_4_ molecules on the investigated 2D nanosheets. Within the FMO analyses, the energies of the highest occupied molecular orbitals (*E*_HOMO_) and lowest unoccupied molecular orbitals (*E*_LUMO_) for the most stable relaxed CX_4_∙∙∙*β*_12_/GN complexes were computed. The energy gap (*E*_gap_) was estimated according to the following equation:

(2) $$E_{gap}=E_{LUMO}-E_{HOMO}$$

The charge transfer of the adsorbed CX_4_ molecules was determined using the Bader charge method [55,64] based on the following equation:

(3) $$Q_{t}=Q_{combined\;2D\;nanosheets}-Q_{isolated\;2D\;nanosheets}$$

where $Q_{combined\;2D\;nanosheets}$ and $Q_{isolated\;2D\;nanosheets}$ are the charges of the 2D nanosheets after adsorbing tetrahalomethanes and the charge of the isolated 2D nanosheets, respectively. The charge density difference (∆*ρ*) was plotted according to the following equation:

(4) $$∆\rho =\rho_{{CX}_{4}\cdot \cdot \cdot 2D\;nanosheet}-\rho_{{CX}_{4}}-\rho_{2D\;nanosheet}$$

where $\rho_{{CX}_{4}\cdot \cdot \cdot 2D\;nanosheet}$, $\rho_{{CX}_{4}}$, and $\rho_{2D\;nanosheet}$ are the charge densities of complex, tetrahalomethane, and 2D nanosheet, respectively. VESTA 3 visualization software was invoked for generating the charge density plots [65]. To comprehend the influence of the adsorption process of the tetrahalomethanes on the electronic characteristics of the *β*_12_ and GN nanosheets, band structure and density of state (DOS) calculations were executed. For implicit water solvent calculations, the Environ code [66] of Quantum ESPRESSO was utilized with a dielectric constant of 78.3. The solvent effect on the adsorption energy of the studied complexes ($E_{ads}^{\mathrm{solvent effect}}$) was computed according to the following equation:

(5) $$E_{ads}^{\mathrm{solvent effect}}=E_{ads}^{water}-E_{ads}^{vacuum}$$

where $E_{ads}^{water}$ and $E_{ads}^{vacuum}$ are the adsorption energies of the complex in water and vacuum media, respectively.

## 4. Conclusions

In the presented work, a DFT study was conducted to comparatively illustrate the adsorption features of tetrahalomethanes (CX_4_, where X = F, Cl, and Br) on *β*_12_ borophene (*β*_12_) and GN nanosheets. To attain a thorough investigation, geometry relaxation, adsorption energies, Bader charge, electronic band structures, and DOS computations were conducted for the adsorption of the CX_4_ molecules on the studied 2D nanosheets within tetrel (XC-X_3_)- and halogen (X_3_C-X)-oriented configurations. From the energetic perspective, the adsorption of the CX_4_ model on the *β*_12_ and GN nanosheets within the tetrel-oriented configuration was more desirable than that within the halogen-oriented configuration. Further favorability of the Br1@*β*_12_ and T@GN adsorption sites were announced toward adsorbing the CX_4_ molecules within the tetrel-oriented configuration and showed the most significant *E*_ads_ for the CBr_4_ molecule with values of −12.33 and −10.03 kcal/mol, respectively. According to the FMO results, the *E*_HOMO_, *E*_LUMO_, and *E*_gap_ values of the *β*_12_ and GN nanosheets were changed following the adsorption process. Based on the Bader charge results, the electron-donating characters for all the CX_4_ molecules after adsorbing on the *β*_12_ and GN nanosheets within tetrel- and halogen-oriented configurations were illustrated, except the CBr_4_∙∙∙GN complexes within the former configuration. In the latter complexes, the adsorbed CBr_4_ molecule showed an electron-accepting property confirmed by the small positive *Q*_t_ values. From the band structure and DOS plots, new bands and peaks were observed, respectively, after the adsorption of CX_4_ molecules on the 2D nanosheets, indicating the occurrence of the adsorption process. The energetic results are pertinent to the solvent effect demonstrated, that the presence of the water solvent led to more observable negative adsorption energies compared with the adsorption in a vacuum. The emerging findings would provide a foundation for any future consideration of *β*_12_ and GN nanosheets to adsorb small molecules.

## Figures and Tables

**Table 1:** Adsorption energies (*E*_ads_, kcal/mol) and equilibrium distances (d, Å) of the relaxed CX_4_∙∙∙*β*_12_/GN complexes (where X = F, Cl, and Br) at all possible sites within the tetrel (XC-X_3_)- and halogen (X_3_C-X)-oriented configurations. Charge transfer difference (*Q*_t_, *e*) for the 2D nanosheets before and after the adsorption process.

2D Nanosheets	Adsorption Site ^a^	X = F			X = Cl			X = Br		
		*E*_ads_ (kcal/mol)	d (Å)	*Q*_t_^b^ (*e*)	*E*_ads_ (kcal/mol)	d (Å)	*Q*_t_^b^ (*e*)	*E*_ads_ (kcal/mol)	d (Å)	*Q*_t_^b^ (*e*)
**Tetrel-oriented configuration**										
***β*** **_12_**	T1	−4.42	3.69	−0.0309	−7.47	4.09	−0.0275	−11.45	4.14	−0.0231
	T2	−4.25	3.72	−0.0289	−7.69	4.06	−0.0230	−11.42	4.13	−0.0080
	T3	−4.14	3.68	−0.0309	−7.22	4.11	−0.0275	−11.07	4.17	−0.0215
	H	−4.39	3.65	−0.0306	−7.21	4.07	−0.0219	−11.15	4.13	−0.0115
	Br1	−4.46	3.67	−0.0313	−7.74	4.05	−0.0283	−12.33	4.11	−0.0263
	Br2	−4.14	3.74	−0.0291	−7.15	4.12	−0.0269	−11.03	4.18	−0.0175
**GN**	T	−4.66	3.50	−0.0175	−7.32	3.93	−0.0072	−10.03	4.07	0.0036
	Br	−4.36	3.57	−0.0177	−6.82	4.02	−0.0051	−9.49	4.13	0.0023
	H	−4.12	3.63	−0.0168	−6.57	4.06	−0.0025	−9.43	4.13	0.0070
**Halogen-oriented configuration**										
***β*** **_12_**	T1	−2.54	3.10	−0.0164	−5.14	3.16	−0.0410	−8.65	3.10	−0.0654
	T2	−2.62	3.12	−0.0149	−4.33	3.26	−0.0254	−6.72	3.21	−0.0291
	T3	−2.63	3.06	−0.0167	−4.97	3.17	−0.0366	--- ^c^	--- ^c^	--- ^c^
	H	−2.71	2.93	−0.0185	−5.58	3.02	−0.0321	−9.00	2.98	−0.0424
	Br1	−2.46	3.11	−0.0165	−5.25	3.13	−0.0413	−8.91	2.98	−0.0697
	Br2	−2.69	3.05	−0.0163	−4.26	3.26	−0.0258	−6.37	3.24	−0.0317
**GN**	T	−2.46	3.00	−0.0095	−4.22	3.16	−0.0149	−6.00	3.17	−0.0196
	Br	−2.47	2.99	−0.0094	−4.18	3.17	−0.0152	−5.93	3.18	−0.0174
	H	−2.61	2.89	−0.0093	−3.99	3.18	−0.0093	−5.54	3.23	−0.0086

^a^ All adsorption sites on the investigated 2D nanosheets are depicted in Figure 2. ^b^ *Q*_t_ was calculated based on Equation (3). ^c^ Desired configuration was not observed after geometry relaxation (see Figure S1).

**Table 2:** The energies of the highest occupied molecular orbitals (*E*_HOMO_, eV), the lowest unoccupied molecular orbitals (*E*_LUMO_, eV), and the energy gap (*E*_gap_, eV) of the CX_4_ molecules and the *β*_12_/GN nanosheets before and after the adsorption process.

Complex ^a^	*E*_HOMO_ (eV)	*E*_LUMO_ (eV)	*E*_gap_ (eV)
**Isolated systems**			
GN Nanosheet	−2.354	−2.343	0.011
*β*_12_ Nanosheet	−2.875	−3.501	−0.626
CF_4_	−10.333	−0.477	9.855
CCl_4_	−7.416	−2.680	4.735
CBr_4_	−6.644	−3.394	3.250
**Tetrel-oriented Configuration**			
FC-F_3_∙∙∙Br1@*β*_12_	−2.734	−3.358	−0.625
ClC-Cl_3_∙∙∙Br1@*β*_12_	−2.602	−3.217	−0.615
BrC-Br_3_∙∙∙Br1@*β*_12_	−2.544	−3.146	−0.602
FC-F_3_∙∙∙T@GN	−2.202	−2.191	0.0104
ClC-Cl_3_∙∙∙T@GN	−2.064	−2.054	0.0107
BrC-Br_3_∙∙∙T@GN	−2.010	−1.999	0.0108
**Halogen-oriented Configuration**			
F_3_C-F∙∙∙H@*β*_12_	−2.737	−3.364	−0.627
Cl_3_C-Cl∙∙∙H@*β*_12_	−2.612	−3.237	−0.626
Br_3_C-Br∙∙∙H@*β*_12_	−2.564	−3.190	−0.626
F_3_C-F∙∙∙H@GN	−2.200	−2.190	0.0104
Cl_3_C-Cl∙∙∙T@GN	−2.066	−2.056	0.0104
Br_3_C-Br∙∙∙T@GN	−2.021	−2.010	0.0105

^a^ Structures of the most stable relaxed CX_4_∙∙∙*β*_12_/GN complexes within both configurations are presented in Figure 3.

**Table 3:** Adsorption energy in the vacuum medium ($E_{ads}^{vacuum}$, kcal/mol), water medium ($E_{ads}^{water}$, kcal/mol), and the energy of the solvent effect ($E_{ads}^{\mathrm{solvent effect}}$, kcal/mol) for the relaxed CX_4_∙∙∙*β*_12_/GN complexes (where X = F, Cl, and Br) at the most preferable adsorption sites within the tetrel (XC-X_3_)- and halogen (X_3_C-X)-oriented configurations.

System ^a^	$E_{ads}^{vacuum}$ (kcal/mol)	$E_{ads}^{water}$ (kcal/mol)	$E_{ads}^{{\mathrm{solvent effect}}^{b}}$ (kcal/mol)
**Tetrel-oriented Configuration**			
FC-F_3_∙∙∙Br1@*β*_12_	−4.46	−6.91	−2.45
ClC-Cl_3_∙∙∙Br1@*β*_12_	−7.74	−11.21	−3.47
BrC-Br_3_∙∙∙Br1@*β*_12_	−12.33	−15.99	−3.66
FC-F_3_∙∙∙T@GN	−4.66	−7.14	−2.48
ClC-Cl_3_∙∙∙T@GN	−7.32	−10.99	−3.67
BrC-Br_3_∙∙∙T@GN	−10.03	−14.09	−4.06
**Halogen-oriented Configuration**			
F_3_C-F∙∙∙H@*β*_12_	−2.71	−4.22	−1.51
ClC-Cl_3_∙∙∙H@*β*_12_	−5.58	−7.26	−1.68
BrC-Br_3_∙∙∙H@*β*_12_	−9.00	−10.60	−1.60
F_3_C-F∙∙∙H@GN	−2.61	−4.17	−1.56
Cl_3_C-Cl∙∙∙T@GN	−4.22	−5.98	−1.76
Br_3_C-Br∙∙∙T@GN	−6.00	−7.72	−1.72

^a^ The structures of the relaxed complexes are depicted in Figure 3. ^b^ $E_{ads}^{\mathrm{solvent effect}}=E_{ads}^{water}-E_{ads}^{vacuum}$.

## Data Availability

Data will be made available on request.

## Supplementary Materials

The following supporting information can be downloaded at: https://www.mdpi.com/article/10.3390/molecules28145476/s1, Figure S1: Side and top representations for the relaxed structures of the tetrel (XC-X_3_)- and halogen (X_3_C-X)-oriented configurations of the CX_4_∙∙∙*β*_12_/GN complexes (where X = F, Cl, and Br) at all the adsorption sites. Equilibrium distances (d) are given in Å; Figure S2: Electronic band structures of *β*_12_ and GN nanosheets along the high symmetry points of the Brillouin zone. The Fermi energy was set at zero energy, and the Dirac point is defined by the dotted circle; Figure S3: Total and projected density of state (TDOS/PDOS) plots for the pure surfaces of *β*_12_ and GN nanosheets, assuming Fermi level as the reference level. The dotted circle defines the Dirac point. The contributions of the *p*-orbital for boron (B) and carbon (C) atoms are represented by B*_p_* and C*_p_*, respectively.

## Sample Availability

Samples of the compounds (XYZ coordinates) are available from the authors.
