# Supplementary material for: Adsorption Features of Tetrahalomethanes (CX4; X = F, Cl, and Br) on β12 Borophene and Pristine Graphene Nanosheets: A Comparative DFT Study
Source: Molecules. 2023 Jul 18;28(14):5476. doi: 10.3390/molecules28145476 (PMC10386295; doi:10.3390/molecules28145476)
Supplement: Supplementary file 1 [file molecules-28-05476-s001.zip › molecules-2491139-supplementary.pdf]

Adsorption Features of Tetrahalomethanes (CX_4; X = F, Cl, and Br) on β_{12} Borophene and Pristine Graphene Nanosheets: A Comparative DFT Study

Mahmoud A. A. Ibrahim[1,2*], Amna H. M. Mahmoud[1], Nayra A. M. Moussa[1], Gamal A. H. Mekhemer[1], Shaban R. M. Sayed[3], Muhammad Naeem Ahmed[4], Mohamed K. Abd El-Rahman[5], Eslam Dabbish[6], and Tamer Shoeib[6*]

[1] Computational Chemistry Laboratory, Chemistry Department, Faculty of Science, Minia University, Minia 61519, Egypt; m.ibrahim@compchem.net (M.A.A.I.); a.mahmoud@compchem.net (A.H.M.M.); n.moussa@compchem.net (N.A.M.M.); gmekhemer@mu.edu.eg (G.A.H.M.)

[2] School of Health Sciences, University of KwaZulu-Natal, Westville, Durban 4000, South Africa

[3] Department of Botany and Microbiology, College of Science, King Saud University, P.O. Box 2455, Riyadh 11451, Saudi Arabia; shmohamed@ksu.edu.sa (S.R.M.S.)

[4] Department of Chemistry, The University of Azad Jammu and Kashmir, Muzaffarabad 13100, Pakistan; drnaeem@ajku.edu.pk (M.N.A.)

[5] Department of Chemistry and Chemical Biology, Harvard University, 12 Oxford Street, Cambridge, MA 02138, USA; kabdelazim@gmwgroup.harvard.edu (M.K.A.E.-R.)

[6] Department of Chemistry, The American University in Cairo, New Cairo11835, Egypt; emoustafa@aucegypt.edu (E.D.); t.shoeib@aucegypt.edu (T.S.)

***** Correspondence: m.ibrahim@compchem.net (M.A.A.I.) and t.shoeib@aucegypt.edu (T.S.).

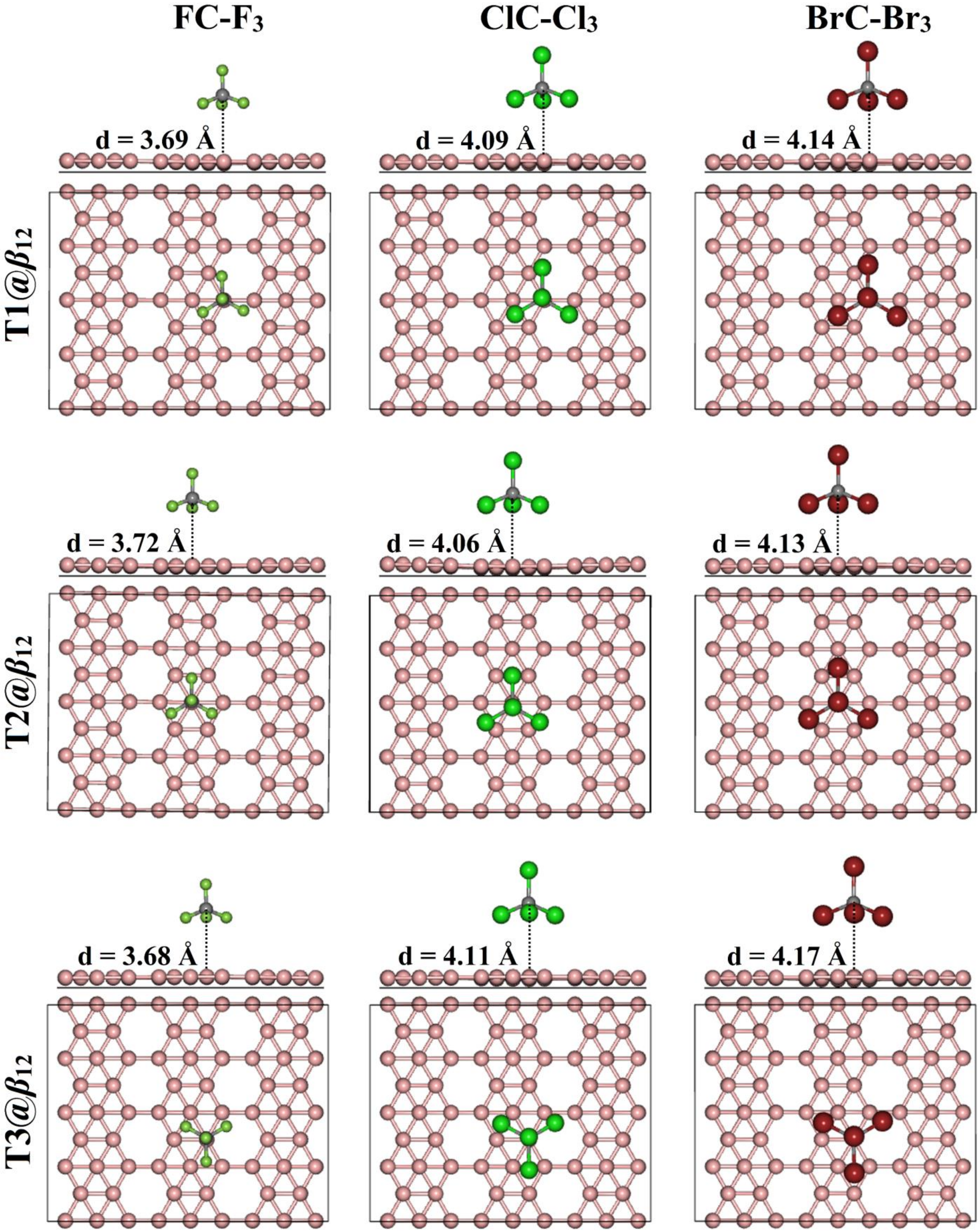

Figure S1. Side and top representations for the relaxed structures of the tetrel (XC-X_3)- and halogen (X_3C-X)-oriented configurations of the $CX_4\cdots\beta_{12}$/GN complexes (where X = F, Cl, and Br) at all the adsorption sites. Equilibrium distances (d) are given in Å.

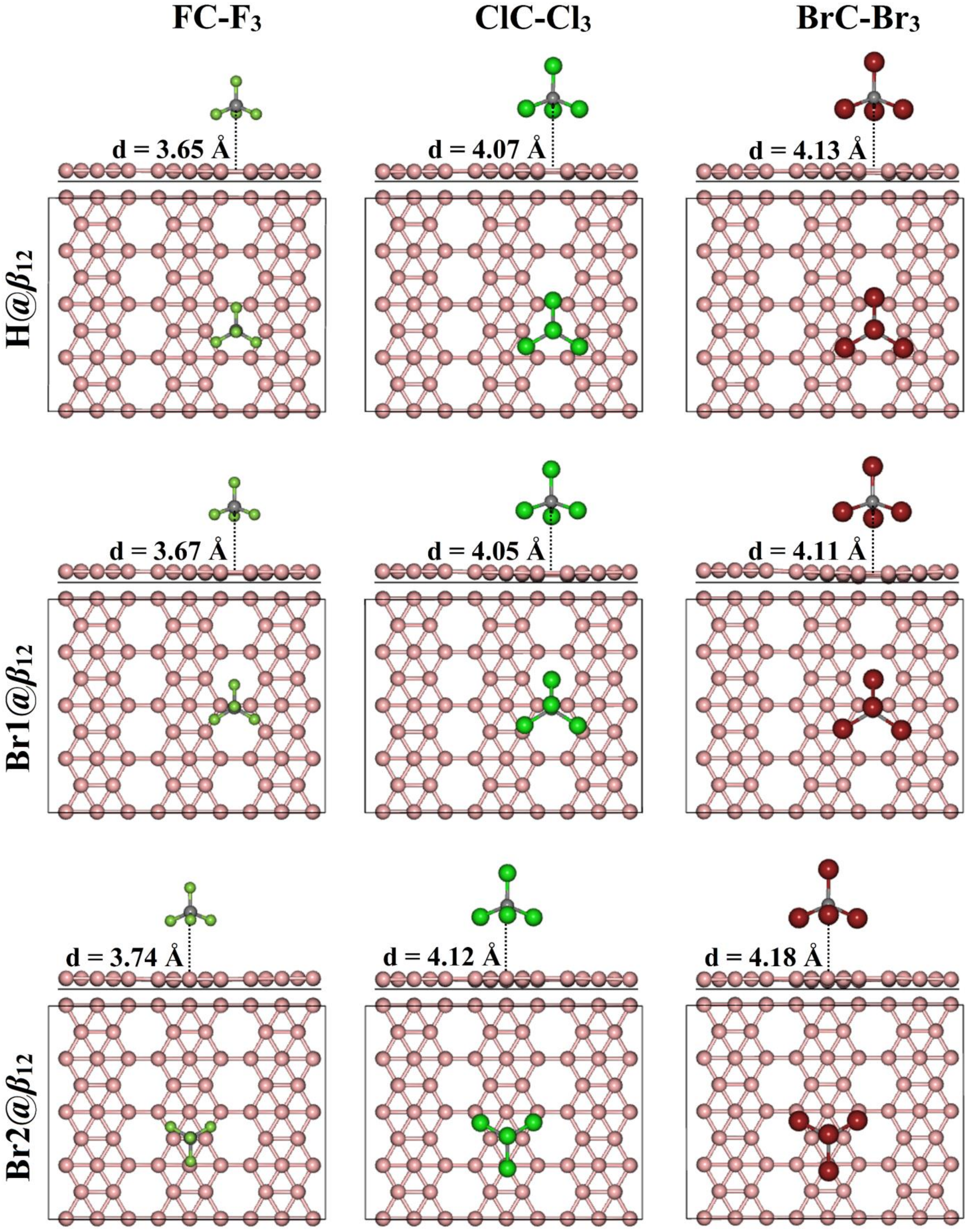

Figure S1. *Continued*.

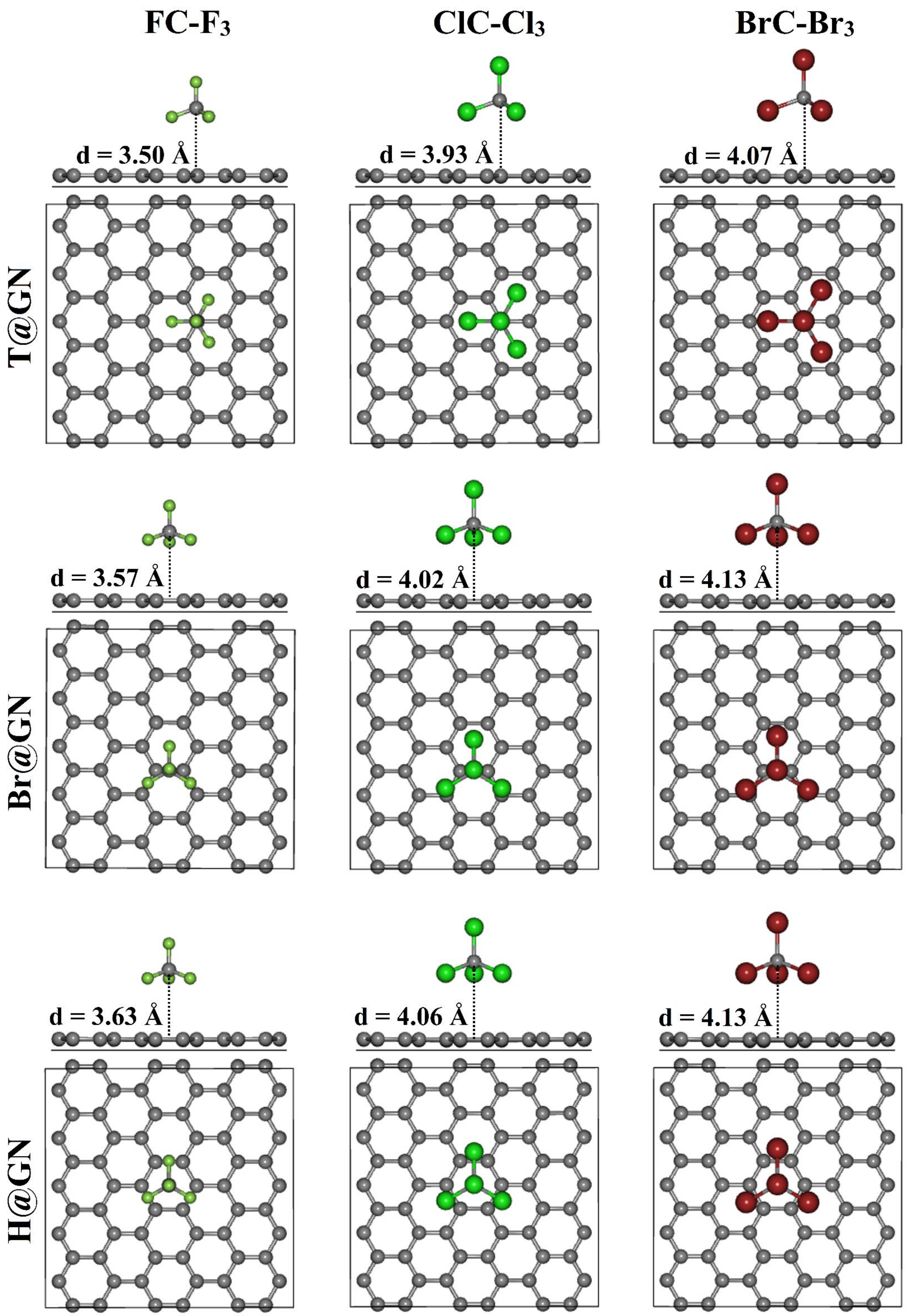

Figure S1. *Continued*.

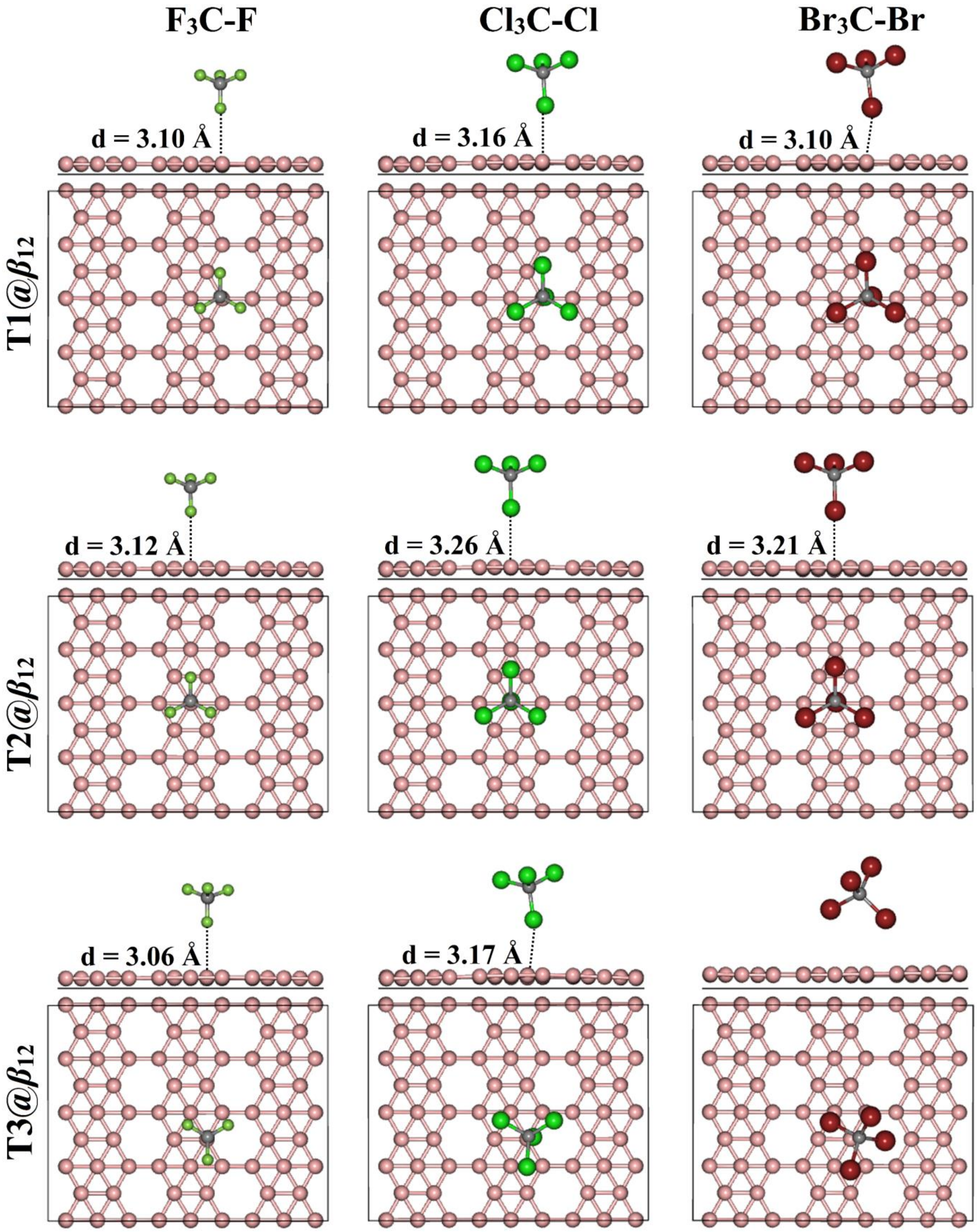

Figure S1. *Continued.*

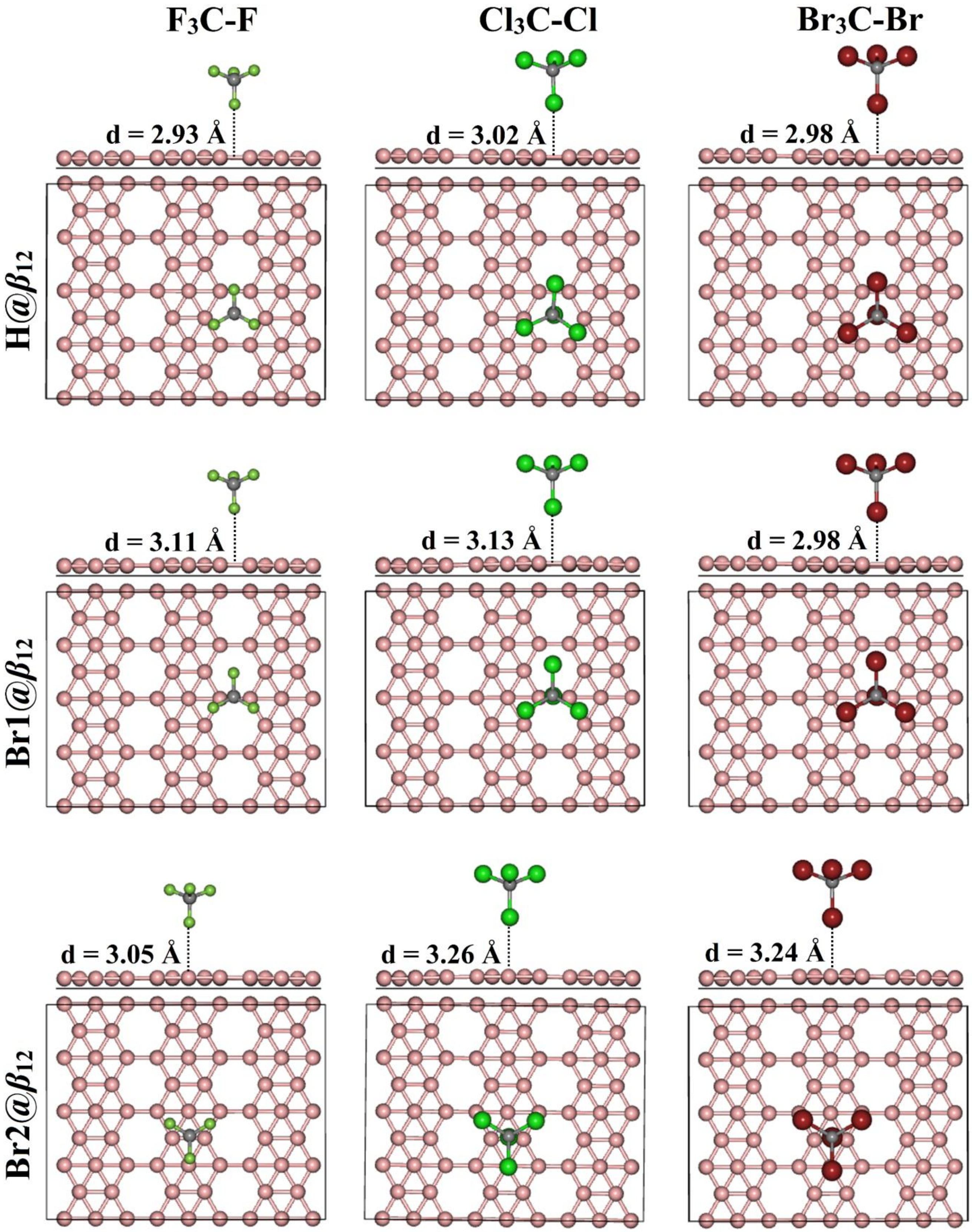

Figure S1. *Continued.*

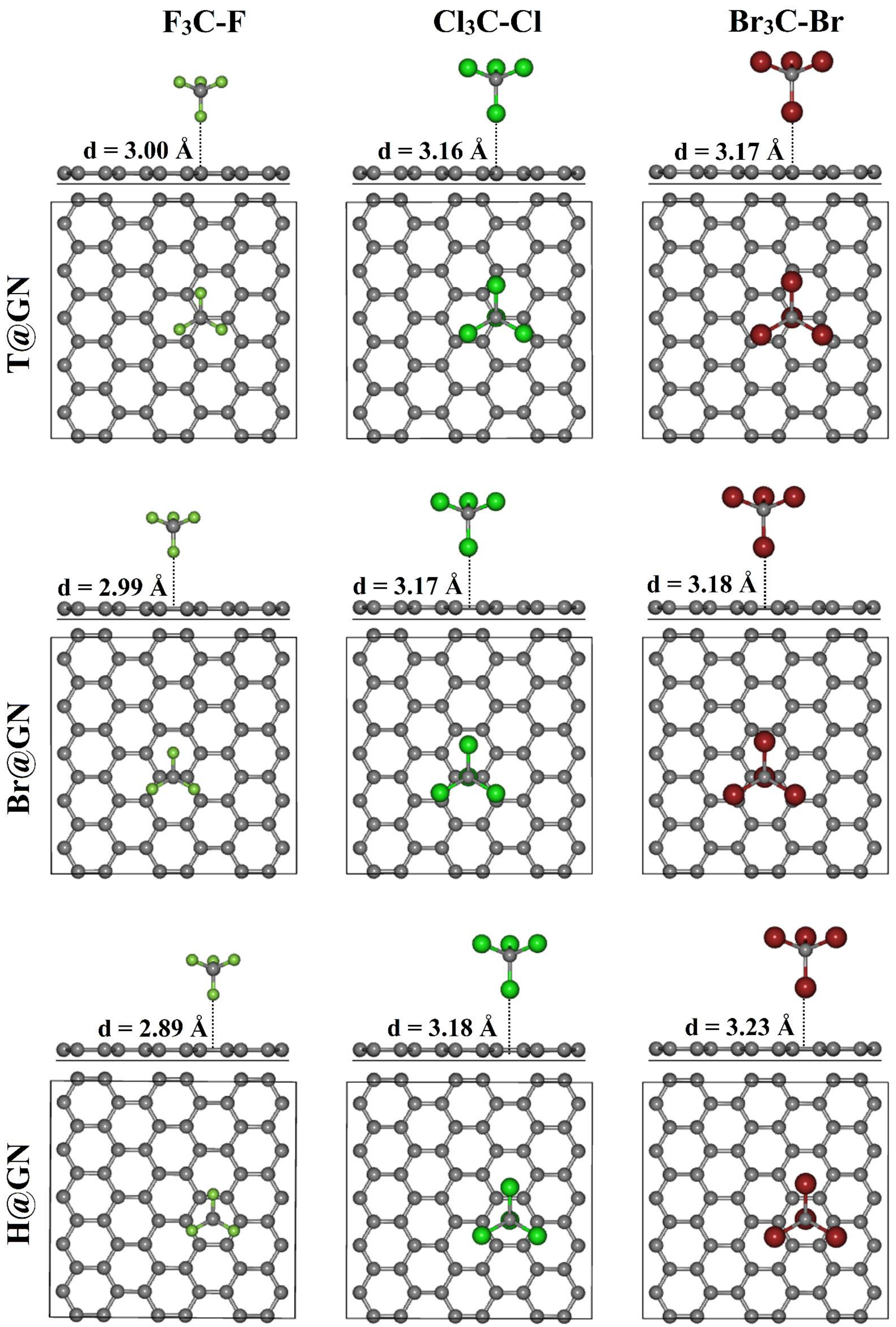

Figure S1. *Continued.*

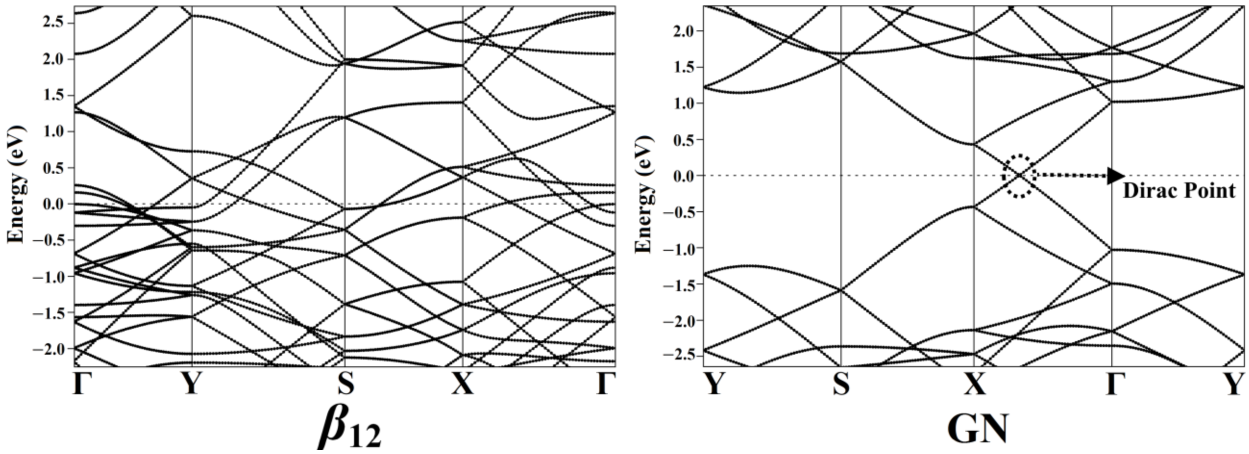

Figure S2. Electronic band structures of β_{12} and GN nanosheets along the high symmetry points of the Brillouin zone. The Fermi energy was set at zero energy, and the Dirac point is defined by the dotted circle.

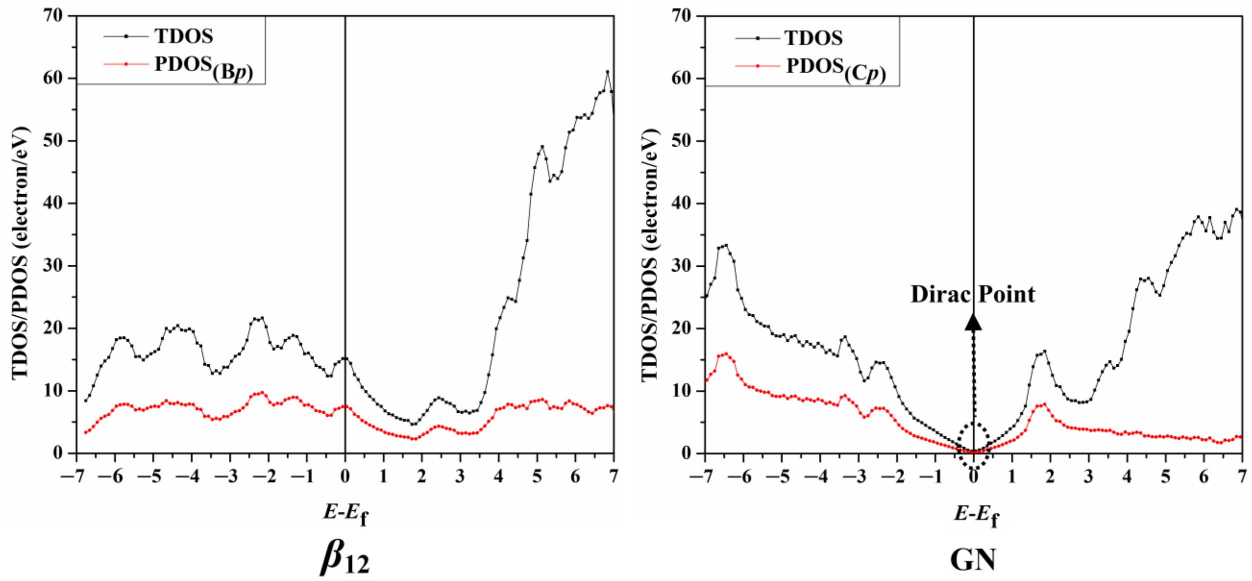

Figure S3. Total and projected density of state (TDOS/PDOS) plots for the pure surfaces of β_{12} and GN nanosheets, assuming Fermi level as the reference level. The dotted circle defines the Dirac point. The contributions of the *p*-orbital for boron (B) and carbon (C) atoms are represented by B_p and C_p, respectively.
